# Supplementary material for: Luqin-like RYamide peptides regulate food-evoked responses in C. elegans
Source: eLife. 2017 Aug 29;6:e28877. doi: 10.7554/eLife.28877 (PMC5576490; doi:10.7554/eLife.28877)
Supplement: Supplementary file 1. [file elife-28877-supp1.docx]

**Supplementary file 1: Results of lifespan analyses (three independent experiments)**

| Experiment 1 | | | | |
| --- | --- | --- | --- | --- |
| Genotype | Mean LS ± SEM (days) | Number of worms | % change versus control | P value versus control |
| *Ex*[*myo-3^prom^::venus*]. (WT) | 16.6 ± 0.4 | 60 | Control | control |
| *npr-22(ok1598) IV*; *Ex*[*myo-3^prom^::venus*]. | 16.8 ± 0.4 | 59 | +1.1 | 0.7993 vs WT |
| *lury-1(gk961835) III*;  *Ex*[*myo-3^prom^::venus*]. | 18.1 ± 0.4 | 61 | +8.8 | 0.0302 vs WT |
| *Ex*[*lury-1(+)*, *myo-3^prom^::venus*]. line #1 | 22.1 ± 0.5 | 77 | +33.0 | < 0.0001 vs WT |
| *npr-22(ok1598) IV*; *Ex*[*lury-1(+)*,  *myo-3^prom^::venus*]. line #1 | 17.6 ± 0.3 | 81 | +5.8 | < 0.0001 vs *Ex*[*lury-1(+)*] |
| *peIs2413*[*lury-1(+)*, *myo-3^prom^::venus*] *II*. | 20.1 ± 0.7 | 59 | +21.1 | < 0.0001 vs WT |
| *peIs2414*[*lury-1(+)*, *myo-3^prom^::venus*]. | 24.9 ± 1.0 | 55 | +50.3 | < 0.0001  vs WT |
| *peIs2413*[*lury-1(+)*, *myo-3^prom^::venus*] *II*;  *npr-22(ok1598) IV*. | 16.9 ± 0.5 | 59 | +1.7 | < 0.0001  vs *pe2413* |
| *npr-22(ok1598) IV*; *peIs2414*[*lury-1(+)*,  *myo-3^prom^::venus*]. | 19.1 ± 0.7 | 59 | +15.2 | < 0.0001  vs *pe2414* |

| Experiment 2 | | | | |
| --- | --- | --- | --- | --- |
| Genotype | Mean LS ± SEM (days) | Number of worms | % change versus control | P value versus control |
| *Ex*[*myo-3^prom^::venus*]. (WT) | 17.8 ± 0.3 | 79 | Control | control |
| *npr-22(ok1598) IV*; *Ex*[*myo-3^prom^::venus*]. | 17.9 ± 0.3 | 91 | +0.7 | 0.7333 vs WT |
| *Ex*[*lury-1(+)*, *myo-3^prom^::venus*]. line #1 | 22.1 ± 0.4 | 95 | +24.3 | < 0.0001 vs WT |
| *Ex*[*lury-1(+)*, *myo-3^prom^::venus*]. line #2 | 22.6 ± 0.4 | 85 | +27.1 | < 0.0001 vs WT |
| *Ex*[*lury-1(+)*, *myo-3^prom^::venus*]. line #3 | 21.7 ± 0.4 | 80 | +22.2 | < 0.0001 vs WT |
| *npr-22(ok1598) IV*;  *Ex*[*lury-1(+)*, *myo-3^prom^::venus*]. line #1 | 17.9 ± 0.3 | 92 | +0.8 | < 0.0001 vs *Ex*[*lury-1(+)*] line #1 |
| *Ex*[*rimb-1^prom^::lury-1*, *myo-3^prom^::venus*]. line #1 | 22.0 ± 0.4 | 91 | +23.7 | < 0.0001  vs WT |
| *Ex*[*rimb-1^prom^::lury-1*, *myo-3^prom^::venus*]. line #2 | 22.8 ± 0.4 | 94 | +28.1 | < 0.0001  vs WT |
| *Ex*[*rimb-1^prom^::lury-1*, *myo-3^prom^::venus*]. line #3 | 22.1 ± 0.5 | 79 | +24.5 | < 0.0001  vs WT |
| *npr-22(ok1598) IV*;  *Ex*[*rimb-1^prom^::lury-1*, *myo-3^prom^::venus*]. line #1 | 17.9 ± 0.3 | 86 | +0.8 | < 0.0001 vs *Ex*[*rimb-1^prom^::lury-1(+)*] line #1 |

| Experiment 3 | | | | |
| --- | --- | --- | --- | --- |
| Genotype | Mean LS ± SEM (days) | Number of worms | % change versus control | P value versus control |
| *peIs2413*[*lury-1(+)*, *myo-3^prom^::venus*] *II*. | 22.6 ± 0.6 | 61 | +31.5 | < 0.0001 |
| *peIs2413*[*lury-1(+)*, *myo-3^prom^::venus*] *II*;  *npr-22(ok1598) IV*. | 17.2 ± 0.4 | 50 | Control | control |
| *peIs2413*[*lury-1(+)*, *myo-3^prom^::venus*] *II*;  *npr-22(ok1598) IV*; *Ex*[*ceh-19^prom^::npr-22a*,  *unc-122^prom^::mCherry*]. | 23.0 ± 0.4 | 82 | +34.3 | < 0.0001 |
| *peIs2413*[*lury-1(+)*, *myo-3^prom^::venus*] *II*;  *npr-22(ok1598) IV*; *Ex*[*cat-1^prom^::npr-22a*,  *unc-122^prom^::mCherry*]. | 18.0 ± 0.4 | 69 | +4.7 | 0.1326 |

LS; lifespan. Statistical analyses were conducted using Log-rank (Mantel-Cox) test.
