# Supplementary material for: Luqin-like RYamide peptides regulate food-evoked responses in C. elegans
Source: eLife. 2017 Aug 29;6:e28877. doi: 10.7554/eLife.28877 (PMC5576490; doi:10.7554/eLife.28877)
Supplement: Supplementary file 2. [file elife-28877-supp2.docx]

| **Supplementary file 2: Strains used in this study** | | |
| --- | --- | --- |
| Strain | Genotype | Source |
| N2 | *C. elegans* wild type | CGC |
| RB1405 | *npr-22(ok1598) Ⅳ*. | CGC ^(a)^ |
| JN2442 | *lury-1(gk961835) Ⅲ*. | CGC ^(a)^ |
| JN2443 | *Ex*[*lury-1^prom^(3,458 bp)::venus*, *unc-122^prom^::mCherry*]. | This study |
| JN2444 | *Ex*[*npr-22^prom^(5,679 bp)::venus*, *unc-122^prom^::mCherry*]. | This study |
| JN2445 | *Ex*[*myo-3^prom^::venus*]. | This study |
| JN2446 | *npr-22(ok1598) Ⅳ*; *Ex*[*myo-3^prom^::venus*]. | This study |
| JN2447 | *lury-1(gk961835) Ⅲ*; *Ex*[*myo-3^prom^::venus*]. | This study |
| JN2448 | *Ex*[*lury-1(+)* genomic DNA (5,689 bp), *myo-3^prom^::venus*]. line #1 | This study ^(b)^ |
| JN2492 | *Ex*[*lury-1(+)* genomic DNA (5,689 bp), *myo-3^prom^::venus*]. line #2 | This study ^(b)^ |
| JN2493 | *Ex*[*lury-1(+)* genomic DNA (5,689 bp), *myo-3^prom^::venus*]. line #3 | This study ^(b)^ |
| JN2449 | *Ex*[*rimb-1^prom^::lury-1(+)*, *myo-3^prom^::venus*]. line #1 | This study ^(b)^ |
| JN2494 | *Ex*[*rimb-1^prom^::lury-1(+)*, *myo-3^prom^::venus*]. line #2 | This study ^(b)^ |
| JN2495 | *Ex*[*rimb-1^prom^::lury-1(+)*, *myo-3^prom^::venus*]. line #3 | This study ^(b)^ |
| JN2450 | *npr-22(ok1598) Ⅳ*; *Ex*[*lury-1(+)* genomic DNA (5,689 bp), *myo-3^prom^::venus*]. | This study ^(b)^ |
| JN2451 | *npr-22(ok1598) Ⅳ*; *Ex*[*rimb-1^prom^::lury-1(+)*, *myo-3^prom^::venus*]. | This study ^(b)^ |
| JN2452 | *Ex*[*lury-1^prom^(3,458 bp)::mCherry*, *glr-2^prom^::venus*]. | This study ^(b)^ |
| JN2453 | *Ex*[*lury-1^prom^(3,458 bp)::mCherry*, *flp-18^prom^::venus*]. | This study |
| JN2454 | *Ex*[*npr-22^prom^::mCherry*, *flp-15^prom^::venus*]. | This study |
| JN2455 | *Ex*[*npr-22^prom^::mCherry*, *ceh-19^prom^::venus*]. | This study |
| JN2456 | *Ex*[*npr-22^prom^::mCherry*, *cat-1^prom^::venus*]. | This study |
| JN2457 | *Ex*[*npr-22^prom^::mCherry*, *slt-1^prom^::Venus*]. | This study |
| JN2458 | *Ex*[*npr-22^prom^::venus*, *ins-1^prom^::mCherry*]. | This study |
| JN2459 | *Ex*[*npr-22^prom^::mCherry*, *npr-1^prom^::venus*]. | This study ^(b)^ |
| JN2413 | *peIs2413*[*lury-1(+)* genomic DNA (5,689 bp), *myo-3^prom^::venus*] *Ⅱ*. | This study ^(a), (b)^ |
| JN2414 | *peIs2414*[*lury-1(+)* genomic DNA (5,689 bp), *myo-3^prom^::venus*]. | This study ^(a), (b)^ |
| JN2415 | *peIs2413*[*lury-1(+)*, *myo-3^prom^::venus*] *Ⅱ*; *npr-22(ok1598) Ⅳ*. | This study ^(b)^ |
| JN2416 | *npr-22(ok1598) Ⅳ*; *peIs2414*[*lury-1(+)*, *myo-3^prom^::venus*]. | This study ^(b)^ |
| JN2460 | *peIs2413*[*lury-1(+)*, *myo-3^prom^::venus*] *Ⅱ*; *npr-22(ok1598) Ⅳ*; *Ex*[*npr-22^prom^::npr-22a*, *unc-122^prom^::mCherry*]. | This study ^(b)^ |
| JN2461 | *peIs2413*[*lury-1(+)*, *myo-3^prom^::venus*] *Ⅱ*; *npr-22(ok1598) Ⅳ*; *Ex*[*npr-22^prom^::npr-22b*, *unc-122^prom^::mCherry*]. | This study ^(b)^ |
| JN2462 | *peIs2413*[*lury-1(+)*, *myo-3^prom^::venus*] *Ⅱ*; *npr-22(ok1598) Ⅳ*; *Ex*[*rimb-1^prom^::npr-22a*, *unc-122^prom^::mCherry*]. | This study ^(b)^ |
| JN2463 | *peIs2413*[*lury-1(+)*, *myo-3^prom^::venus*] *Ⅱ*; *npr-22(ok1598) Ⅳ*; *Ex*[*ceh-19^prom^::npr-22a*, *unc-122^prom^::mCherry*]. | This study ^(b)^ |
| JN2464 | *peIs2413*[*lury-1(+)*, *myo-3^prom^::venus*] *Ⅱ*; *npr-22(ok1598) Ⅳ*; *Ex*[*ins-1^prom^::npr-22a*, *unc-122^prom^::mCherry*]. | This study ^(b)^ |
| JN2465 | *peIs2413*[*lury-1(+)*, *myo-3^prom^::venus*] *Ⅱ*; *npr-22(ok1598) Ⅳ*; *Ex*[*ges-1^prom^::npr-22a*, *unc-122^prom^::mCherry*]. | This study ^(b)^ |
| JN2466 | *peIs2413*[*lury-1(+)*, *myo-3^prom^::venus*] *Ⅱ*; *npr-22(ok1598) Ⅳ*; *Ex*[*cat-1^prom^::npr-22a*, *unc-122^prom^::mCherry*]. | This study ^(b)^ |
| JN2467 | *peIs2413*[*lury-1(+)*, *myo-3^prom^::venus*] *Ⅱ*; *npr-22(ok1598) Ⅳ*; *Ex*[*flp-15^prom^::npr-22a*, *unc-122^prom^::mCherry*]. | This study ^(b)^ |
| JN2468 | *peIs2413*[*lury-1(+)*, *myo-3^prom^::venus*] *Ⅱ*; *npr-22(ok1598) Ⅳ*; *Ex*[*myo-2^prom^::npr-22a*, *unc-122^prom^::mCherry*]. | This study ^(b)^ |
| JN2469 | *peIs2413*[*lury-1(+)*, *myo-3^prom^::venus*] *Ⅱ*; *npr-22(ok1598) Ⅳ*; *Ex*[*myo-3^prom^::npr-22a*, *unc-122^prom^::mCherry*]. | This study ^(b)^ |
| JN2470 | *peIs2413*[*lury-1(+)*, *myo-3^prom^::venus*] *Ⅱ*; *npr-22(ok1598) Ⅳ*; *Ex*[*slt-1^prom^::npr-22a*, *unc-122^prom^::mCherry*]. | This study ^(b)^ |
| JN2471 | *peIs2413*[*lury-1(+)*, *myo-3^prom^::venus*] *Ⅱ*; *npr-22(ok1598) Ⅳ*; *Ex*[*unc-122^prom^::mCherry*]. | This study ^(b)^ |
| CB1111 | *cat-1(e1111) X*. | CGC |
| CB1112 | *cat-2(e1112) Ⅱ*. | CGC |
| MT15434 | *tph-1(mg280) Ⅱ*. | From M. Alkema |
| JN2472 | *peIs2413[lury-1(+)*, *myo-3^prom^::venus*] *Ⅱ*; *cat-1(e1111) X*. | This study ^(b)^ |
| JN2473 | *cat-2(e1112)* *peIs2413*[*lury-1(+)*, *myo-3^prom^::venus*] *Ⅱ*. | This study ^(b)^ |
| JN2474 | *tph-1(mg280)* *peIs2413*[*lury-1(+)*, *myo-3^prom^::venus*] *Ⅱ*. | This study ^(b)^ |
| MT9772 | *mod-5(n3314) Ⅰ*. | CGC |
| MT8944 | *mod-5(n822) Ⅰ*. | CGC |
| MT2426 | *goa-1(n1134) Ⅰ*. | CGC ^(a)^ |
| NM1380 | *egl-30(js126) Ⅰ*. | CGC |
| KG571 | *eat-16(ce71) Ⅰ*. | CGC |
| MT6129 | *egl-19(n2368) Ⅳ*. | CGC |
| JN2475 | *Ex*[*lury-1^prom^(3,458 bp)::lury-1(with intron)::venus*]. | This study |
| JN2476 | *Ex*[*flp-7(+)* genomic DNA (4,981 bp), *myo-3^prom^::venus*]. | This study ^(b)^ |
| JN2477 | *mod-5(n3314) I*; *peIs2413*[*lury-1(+)*, *myo-3^prom^::venus*] *Ⅱ*. | This study ^(b)^ |
| JN2478 | *mod-5(n822) I*; *peIs2413*[*lury-1(+)*, *myo-3^prom^::venus*] *Ⅱ*. | This study ^(b)^ |
| JN2479 | *mod-5(n3314)* *Ⅰ*; *peIs2413*[*lury-1(+)*, *myo-3^prom^::venus*] *Ⅱ*; *Ex*[*unc-122^prom^::mCherry*]. | This study ^(b)^ |
| JN2480 | *peIs2413*[*lury-1(+)*, *myo-3^prom^::venus*] *Ⅱ*; *npr-22(ok1598)* *Ⅳ*; *Ex*[*unc-122^prom^::mCherry*]. | This study ^(b)^ |
| JN2481 | *npr-22(ok1598)* *Ⅳ*; *Ex*[*npr-22^prom^::FTF::npr-22a::SL2::mCherry*, *cat-1^prom^::FLP*, *lin-44^prom^::gfp*]. | This study |
| JN2482 | *npr-22(ok1598)* *Ⅳ*; *Ex*[*ceh-19^prom^::FTF::npr-22a::SL2::mCherry*, *npr-22^prom^::FLP*, *lin-44^prom^::gfp*]. | This study |
| JN2484 | *Ex*[*npr-22^prom^::mCherry*, *acr-5^prom^::venus*]. | This study |
| JN2485 | *peIs2413*[*lury-1(+)*, *myo-3^prom^::venus*] *Ⅱ*; *npr-22(ok1598)* *Ⅳ*; *Ex*[*acr-2^prom^::npr-22a*, *unc-122^prom^::mCherry*]. | This study ^(b)^ |
| JN2486 | *peIs2413*[*lury-1(+)*, *myo-3^prom^::venus*] *Ⅱ*; *npr-22(ok1598)* *Ⅳ*; *Ex*[*npr-22^prom^::FTF::npr-22a::SL2::mCherry*, *cat-1^prom^::FLP*, *lin-44^prom^::gfp*]. | This study ^(b)^ |
| JN2487 | *peIs2413*[*lury-1(+)*, *myo-3^prom^::venus*] *Ⅱ*; *npr-22(ok1598)* *Ⅳ*; *Ex*[*ceh-19^prom^::FTF::npr-22a::SL2::mCherry*, *npr-22^prom^::FLP*, *lin-44^prom^::gfp*]. | This study ^(b)^ |
| JN2488 | *peIs2413*[*lury-1(+)*, *myo-3^prom^::venus*] *Ⅱ*; *npr-22(ok1598)* *Ⅳ*; *Ex*[*npr-22^prom^::FTF::npr-22a::SL2::mCherry*, *lin-44^prom^::gfp*]. | This study ^(b)^ |
| a: These strains were outcrossed to N2 four to eight times in our lab before use. | | |
| b: In these strain, *lury-1(+)* (genomic DNA), *flp-7(+)* (genomic DNA), *rimb-1^prom^::lury-1(+)*, *glr-2^prom^::venus*, or *npr-1^prom^::venus* was expressed from linear vector-free DNA. | | |
| Unpublished strains from other labs are not shown here. | | |
